# Brevilin A induces ROS-dependent apoptosis and suppresses STAT3 activation by direct binding in human lung cancer cells

**DOI:** 10.7150/jca.40983

**Published:** 2020-04-06

**Authors:** Muhammad Khan, Amara Maryam, Muhammad Zubair Saleem, Hafiz Abdullah Shakir, Javed Iqbal Qazi, Yongming Li, Tonghui Ma

**Affiliations:** 1 Department of Zoology, University of the Punjab, Quaid-e-Azam Campus, Lahore-54590, Pakistan.; 2 College of Basic Medical Sciences, Dalian Medical University, Dalian-116044, P.R. China; 3 School of Medicine and Holistic Integrative Medicine, Nanjing University of Chinese Medicine, Nanjing 210023, P.R. China

**Keywords:** Brevilin A, NSCLC, ROS, STAT3, SPR, apoptosis

## Abstract

Sesquiterpene lactones have been shown to be promising leads for anticancer drug development. Brevilin A (BLN-A), a sesquiterpene lactone compound of *Centipeda minima* has been shown to exhibit anticancer effects against various cancer cells. However, the anticancer mechanism and cellular targets of BLN-A remain elusive. Here in this study, BLN-A inhibits proliferation and induces cell morphological changes in A549 and NCI-H1650 non-small cell lung cancer cells in a dose-dependent manner. Moreover, BLN-A increased ROS generation and bax/bcl-2 ratio while decreased intracellular glutathione (GSH), and mitochondrial membrane potential which resulted in induction of apoptosis as evident by annexin-V/FITC staining, caspase-3 activation and PARP cleavage. Supplementation of cells with NAC (ROS Scavenger) effectively protected the cells from BLN-A-induced apoptosis. Finally, BLN-A inhibited constitutive as well as IL-6- and EGF-induced STAT3 activation at Tyr705. Using molecular docking and SPR analyses, we found that BLN-A directly binds with STAT3 and thereby inhibits its activation. Knocking down of STAT3 by stable transfection with shRNA suppressed growth and augmented cytotoxicity of BLN-A, indicating the key role of STAT3 in BLN-A-mediated apoptosis. Cumulative findings suggest that BLN-A is a promising lead structure for developing it into a potent STAT3 inhibitor and therapeutic agent against NSCLC as well.

## Introduction

Lung cancer is the most common and most devastating cancer in both genders and represents the first leading cause of cancer-related deaths worldwide 1,2. Non-small cell lung carcinoma (NSCLC) which is common in both smokers and non-smokers 3, is the most prevalent cancer and accounts for ~80-85% of all lung cancer cases 2. Despite recent advancements in surgical therapy, radiation therapy and development of highly targeted drugs such as monoclonal antibodies and receptor tyrosine kinase (RTK) inhibitors, the prognosis of NSCLC remains dismal, with 5 year survival rate about 15% 1,4. Although initially targeted therapies substantially improved the survival rate of patients with NSCLC, these drugs failed to eradicate cancer due to development of secondary drug resistance 1,5,6. Therefore, it is imperative to explore novel and potent therapeutic agents with multiple cellular targets, which in addition to exhibiting potent anticancer activity, could potentially suppress the signaling pathways vital for the development of secondary drug resistance in NSCLC.

Signal transducer and activator of transcription 3 (STAT3) is a transcriptional factor which is activated by phosphorylation at tyrosine 705 (Tyr705) in response to growth factors and cytokines and is implicated in a wide range of cellular functions such as cell proliferation, survival, inflammation, metabolism, and immunity 7, 8. Unlike normal cells where activation of STAT3 is strictly regulated, in cancer cells, it is constitutively activated 2. An increasing body of research evidence has clearly established the functional role of constitutive STAT3 activation in tumor induction, progression and drug resistance in multiple human cancers including NSCLC 1,2,9,10. Since multiple oncogenic pathways converge on STAT3, inhibition of STAT3 activation could simultaneously inhibit several up-stream signaling pathways 10. These findings validated STAT3 as an attractive therapeutic cellular target for anticancer drug discovery. It has recently been reported that conventional therapies for lung cancer including chemotherapeutics and targeted therapies induce STAT3 activation 1,11,12, suggesting that STAT3 might be implicated in development of secondary drug resistance against conventional therapies. Until now, numerous STAT3 inhibitors have been discovered and characterized, however; none of them has been approved by FDA for clinical use in the treatment of cancer 8,13, suggesting that mere inhibition of STAT3 is not sufficient enough to effectively eradicate cancer cells. It is therefore, necessary to identify novel therapeutic agents that could simultaneously suppress STAT3 signaling and trigger apoptosis through different mechanisms. The basic principle of targeting two or more signaling pathways simultaneously relies on the notion that at some point tumor cells will not be able to cope with stress, induced as a result of loss of two distinct survival signalings and will ultimately undergo apoptosis. It is therefore, believed that novel bioactive molecules possessing potent anticancer and anti-STAT3 activity could effectively set the cancer cells on the road to ruin and could be potentially developed into novel anti-cancer drugs.

Sesquiterpene lactones are a family of naturally occurring small molecule compounds with multiple medicinal effects in different human ailments including inflammation and cancer 14, 15. Brevilin A (BLN-A), is a sesquiterpene lactone constituent of *Centipeda minima* herb. BLN-A has been investigated for its anti-growth and anticancer activities in only a few cancer cell lines 14,16,17. Moreover, we and Chen et al., have previously reported that BLN-A suppresses STAT3 activation 14, 17, however; the in-depth mechanism by which BLN-A inhibits STAT3 signaling and induces apoptosis remains elusive. The present study was undertaken to delve the anticancer mechanism of BLN-A using lung adenocarcinoma cells. Moreover, the present study demonstrates the in-depth molecular mechanism of anti-STAT3 activity of BLN-A and functional role of STAT3 in BLN-A-induced apoptosis in A549 lung cancer cells.

## Materials and Methods

### Cell lines, reagents and antibodies

Human non-small cell lung carcinoma cell lines (A549 and NCI-H1650) were obtained from Shanghai Cell Bank (China). STAT3 knockdown A549 cell line was previously generated 1. BLN-A (purity >98%) was obtained from Dalian Meilum Biotechnology, Co., Ltd. Cell culture medium and FBS were purchased from Gibco. All the antibodies (primary & secondary) were purchased from Cell Signaling Technology. STAT3 inhibitor (S31-201) and JAKs inhibitor momelotinib (MLT) were purchased from Selleckchem (Munich, Germany). All other reagents and kits were purchased from Beyotime (Nanjing, China) unless otherwise stated.

### Determination of cytotoxicity of BLN-A

Cytotoxicity of BLN-A was assessed by MTT assay and observing cell morphological changes. For MTT assay, lung cancer cells were treated in triplicate with 0, 5, 15, 30, 60 and 100 µM BLN-A in 96 well plates for 12 h. Control cells were treated with 0.5µL DMSO. After 12 h, MTT assay was performed to calculate cell viability as described by us previously 18^.^ Phase-contrast microscope (Leica DMIL LED) was used to monitor cells morphology. Images of control and treated cells were captured with DCF450C camera equipped with microscope.

### Determination of apoptosis

Apoptosis was determined using apoptosis assay kit (Beyotime). Briefly, A549 cells were treated with 0, 20, 30, 40, and 50 µM BLN-A for 12 h. NAC was used as ROS scavenger. After 12 h drug treatment, cells were harvested by centrifugation. The cell pallets were washed with cold PBS and resuspended in 500µL binding buffer. The samples were further incubated with 5 µL Annexin V and 10 µL PI for 15 min in the dark. Finally, the samples were washed to remove extra stain and analyzed by BD Accuri C^6^ flow cytometery for apoptosis rates.

### Determination of ROS production and mitochondrial membrane potential (MMP)

Intracellular ROS production in control and drug treated cells was measured using ROS assay kit (Beyotime). MMP in control and BLN-A treated cells was determined using MMP assay kit (Beyotime). The detailed protocols for measurement of ROS and MMP were described by us previously 2.

### Measurement of reduced glutathione (GSH)

The intracellular GSH level was measured using a GSH-reduced assay kit (Sigma-Aldrich). Briefly, the cells were seeded in 6 well plates in triplicate and incubated overnight in CO_2_ incubator. The cells were harvested after 12 h BLN-A post-treatment and samples were prepared for GSH measurement. The GSH contents were determined following the kit's instructions.

### Trypan blue dye exclusion (TBE) assay

For TBE assay, the cells were exposed to BLN-A treatment in 6 well plates for 12 h. The cell pellets were obtained by harvesting both adherent and floating cells. Finally, cell pellets were washed and resuspended in 200 µL PBS. After adding 200 µL of 0.4% trypan blue solution into cells suspension, the samples were incubated for 5 min at room temperature. Following incubation with trypan blue, the samples were centrifuged and cell pellets were washed to remove extra stain. After washing, the cell pellets were resuspended in PBS and observed under microscope. Dead cells take up trypan blue and appear blue under microscope. For dead cells quantification, three different regions were selected randomly and 100 cells were counted from each region.

### Colony forming assay

A549 cells (scrambled shRNA treated and STAT3 shRNA transfected) were treated with BLN-A for 12 h. STAT3 knockdown A549 cell line was previously described 1. After BLN-A treatment, dead cells (floating cells) were removed while adherent cells were washed with PBS and collected using trypsin. Subsequently, 300 cells were plated into 6 well plates and incubated in CO_2_ incubator for 10 days. The colonies were observed microscopically and stained with crystal violet solution after fixing with 4% PFA. The images were captured after washing the colonies to remove extra stain from the wells. For quantification of proliferation rate, crystal violet stain was dissolved by adding 500µL methanol to each well. At the end, 100 µL solution was transferred into 96 well plates in triplicate and absorbance was measured at 595 nm.

### STAT3 DNA binding Assay

STAT3 DNA binding activity was determined using commercially available TransAM ^TM^ STAT3 assay kit (Active motif Inc, Carlsbad, CA). Briefly, after overnight incubation in CO_2_ incubator, the cells were incubated with BLN-A (0, 20 and 30 µM) for 4 h. After 4 h drug treatment, the cells were stimulated with IL-6 for 30 min. Finally, nuclear extracts were prepared and subjected for STAT3 DNA binding activity according to the kit instructions.

### Western blot analysis

Total proteins from control and drug treated samples were extracted using RIPA buffer as described previously 2. Following measurement of protein concentration with BCA kit, protein samples were denatured by mixing equal amount of 2x loading buffer at 100°C for 5 min. Finally, 40µg proteins were loaded on 10% SDS-PAGE, and immunoblotting was performed as described by us previously 1, 2.

### Molecular docking

Molecular docking was employed to predict the binding interactions between STAT3 and BLN-A. LIGPLOT and PyMOL (v 1.3) programs were used to perform molecular docking as described by us previously 1.

### Surface Plasma Resonance (SPR) analysis

Recombinant human STAT3 protein was purchased from abcam (Cambridge, MA). The SPR analysis was performed as described previously 19. Briefly, to test BLN-A binding with STAT3, a series a BLN-A concentrations from 19.53nM to 5000nM were tested on C5 sensor chip immobilized with STAT3. BLN-A was injected at a flow rate of 30µL/min. The association time was set to 90.1 sec and dissociation time was set to 90.4 sec.

### Statistical analysis

Graphpad Prism 6.0 was used to analyze data. Student's t-test was applied to compare means when only two groups were involved in the study. For multiple group comparison, One-Way ANOVA with Tukey's multiple comparison test was used. Values are presented as Mean±SEM (n=3). P<0.05 was considered statistically significant.

## Results

### BLN-A inhibits growth and promotes apoptosis in lung adenocarcinoma cells

**Figure 1:**
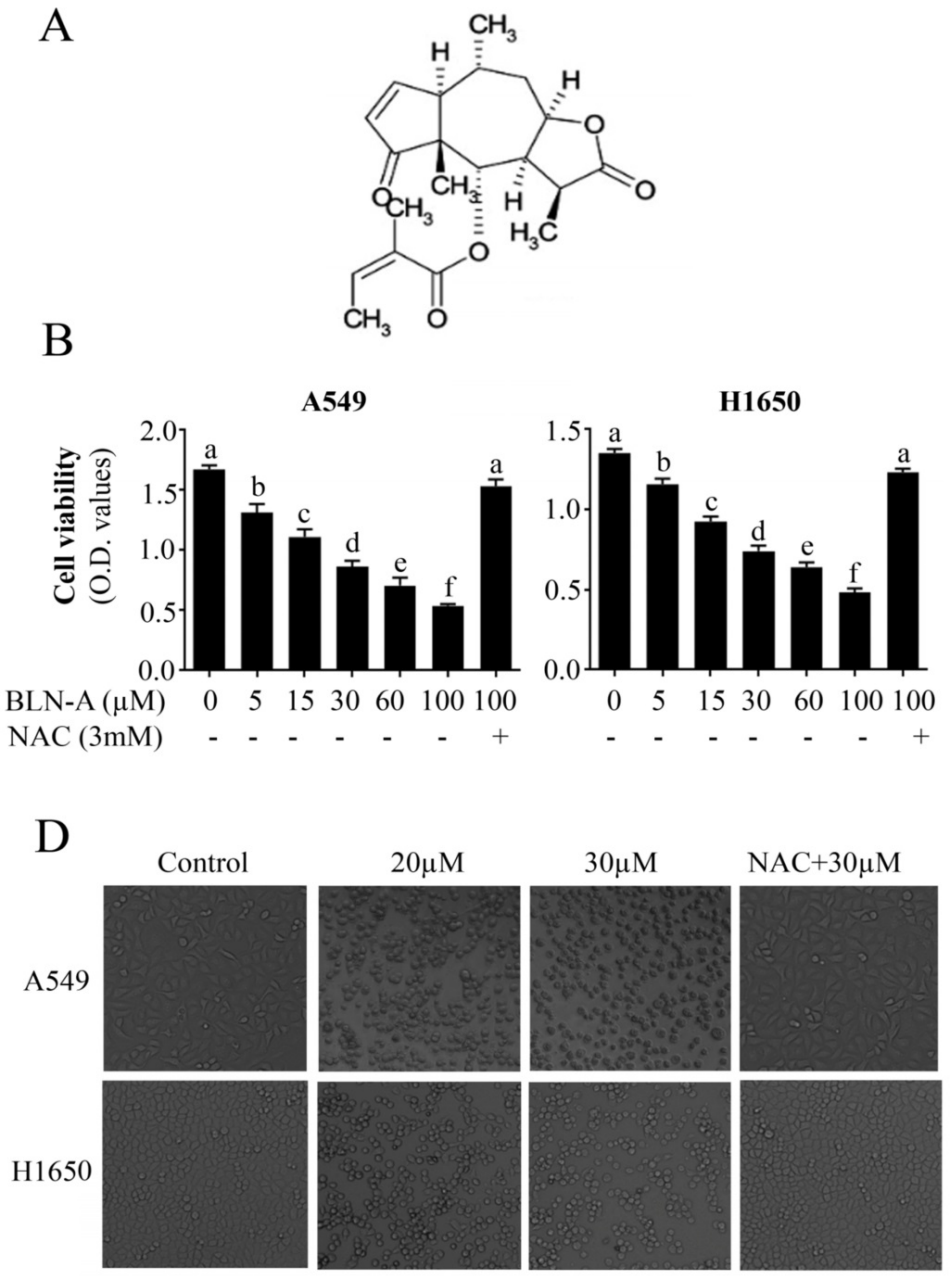
Effect of BLN-A on cell viability and cell morphology. **(A)** Chemical structure of BLN-A. **(B)** A549 and H1650 cells were incubated with BLN-A in triplicates for 12 h. To evaluate the effect of ROS, NAC was added 30 minutes before the drug treatment. Following treatments, MTT assay was performed to measure cell viability. **(C)** After drug treatment, cells morphological changes were observed by phase-contrast microscope.

The growth inhibitory activity of BLN-A in lung adenocarcinoma cells was measured by MTT assay using A549 and NCI-H1650 cells. BLN-A halted the growth of both cell lines in a dose-dependent manner (Figure 1B). The anti-proliferative effect of BLN-A against both cell lines was comparable. The cytotoxicity of BLN-A was further evaluated by observing cell morphological changes. Treatment of both cell lines with BLN-A for 12 h resulted in induction of drastic changes in cells morphology which are characteristically linked with cell death such as rounding up of cells, loss of anchorage of cells with culture plates, change in normal cell geometry and presence of floating cells in culture medium (Figure 1B). However, pretreatment of cells with antioxidant N-acetyl-cysteine (NAC) completely suppressed the anti-proliferative and cytotoxic effects of BLN-A in both cancer cell lines indicating that these effects of BLN-A are mediated through induction of oxidative stress (Figure 1A & B). Since the anticancer activity of BLN-A was comparable in both cell lines; we selected a single cell line (A549 cell line) for further mechanistic study.

**Figure 2:**
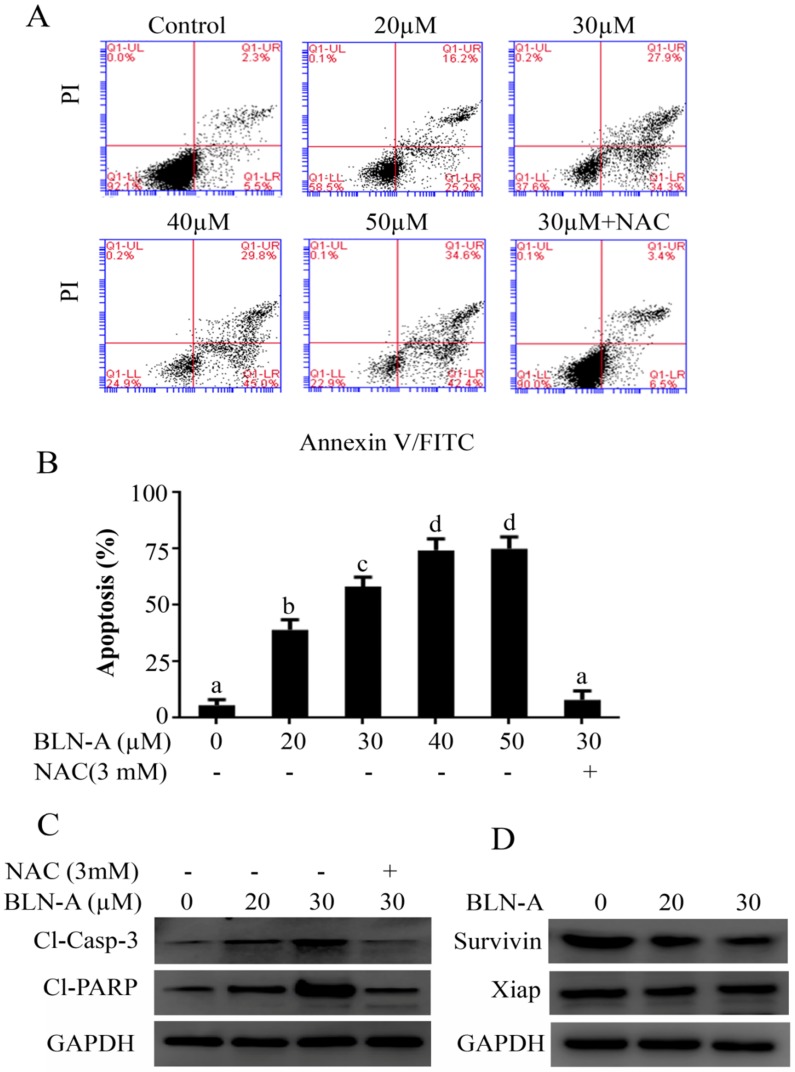
Determination of apoptosis inducing effect of BLN-A. The control and BLN-A treated Cells were harvested and subjected V-FITC/PI staining using apoptosis assay kit as per manufacturer's instructions. The samples were analyzed by flow cytometry. **(B)** Data obtained by FCM was quantified and expressed as mean±SEM (n=3). Columns with different superscript letters differ significantly (p<0.05). **(C)** Cell lysates were prepared after exposing the cells with BLN-A as indicated and expression of cleaved-caspase-3, cleaved PARP, survivin and xiap were measured using Western blots. The quantification of immunoblots in the form of bar-graph has been shown in supplementary Figure S1 (A & B).

To characterize the nature of BLN-A mediated anticancer activity, we performed apoptosis assay using flow cytometry. The data demonstrated that BLN-A treatment increased apoptosis dose-dependently. The maximum apoptotic effect was achieved at a dose of 40 µM. The apoptotic nature of cell death was further verified by detecting the expression of cleaved caspase-3 and cleaved PARP by immunoblotting, which are the classical markers of apoptosis. We observed an increased expression of cleaved caspase-3 and cleaved PARP in cells treated with BLN-A. In agreement with the data of Figure 1, NAC pretreatment completely protected the cells from apoptotic potential of BLN-A as evident from Figure 2A and C. In addition, we also examined the expression of Xiap and survivin which are negative regulators of apoptosis. BLN-A inhibited the expression of survivin, however; the expression of Xiap remained unchanged (Figure 2D).

### BLN-A induces oxidative stress, disrupts MMP and modulates Bcl-2 family proteins

**Figure 3:**
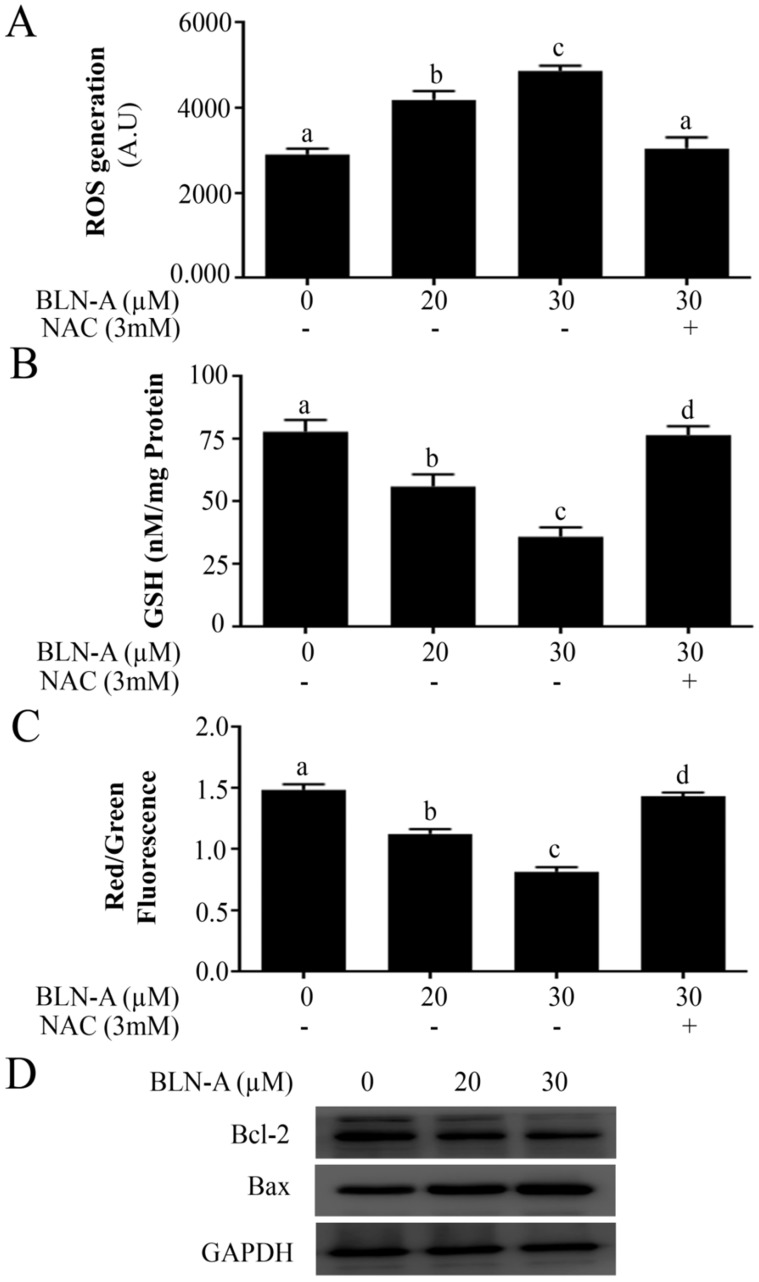
Effect of BLN-A on ROS and GSH level, and MMP and Bcl-2 family proteins expression. **(A, B)** Cells were exposed with BLN-A for 4 h. NAC was added 30 min before drug treatment. Samples were prepared and ROS generation (A) and intracellular GSH (B) were measured using commercially available kits. (**C**) Cells were exposed to BLN-A for 12 h as indicated and MMP was measured using JC-1 kit as per manufacturer's instructions. Columns with different superscript letters differ significantly (p<0.05) from each other (A, B and C). **(D)** Cellular extracts were prepared from control and BLN-A treated samples and expressions of Bcl-2 and Bax were determined by immunoblotting. The quantification of Western blot images in the form of bar-graph has been shown in supplementary Figure S1 (C).

Since BLN-A-induced apoptosis was suppressed by NAC, we hypothesized that BLN-A induces apoptosis in A549 cells through a mechanism which involves oxidative stress. To test our hypothesis, we treated the cells with BLN-A and measured the level of intracellular ROS and reduced glutathione (GSH). Our results showed that BLN-A increased the intracellular ROS generation and decreased intracellular GSH in A549 cells in a dose-dependent manner (Figure 3A and B). Next we measured MMP and data demonstrated that BLN-A caused a dose-dependent decrease in MMP (Figure 3C). However, all these events were reversed when cells were pretreated with NAC, indicating that BLN-A mainly triggers its effects via induction of oxidative stress. Moreover, BLN-A decreased Bcl-2/Bax ratio in A549 cells in a dose-dependent manner (Figure 3D). Both these events (MMP dissipation and decrease in Bcl-2/bax ratio) indicate that BLN-A mediated apoptosis is associated with mitochondrial apoptosis.

### BLN-A inhibits constitutive and inducible activation of STAT3

**Figure 4:**
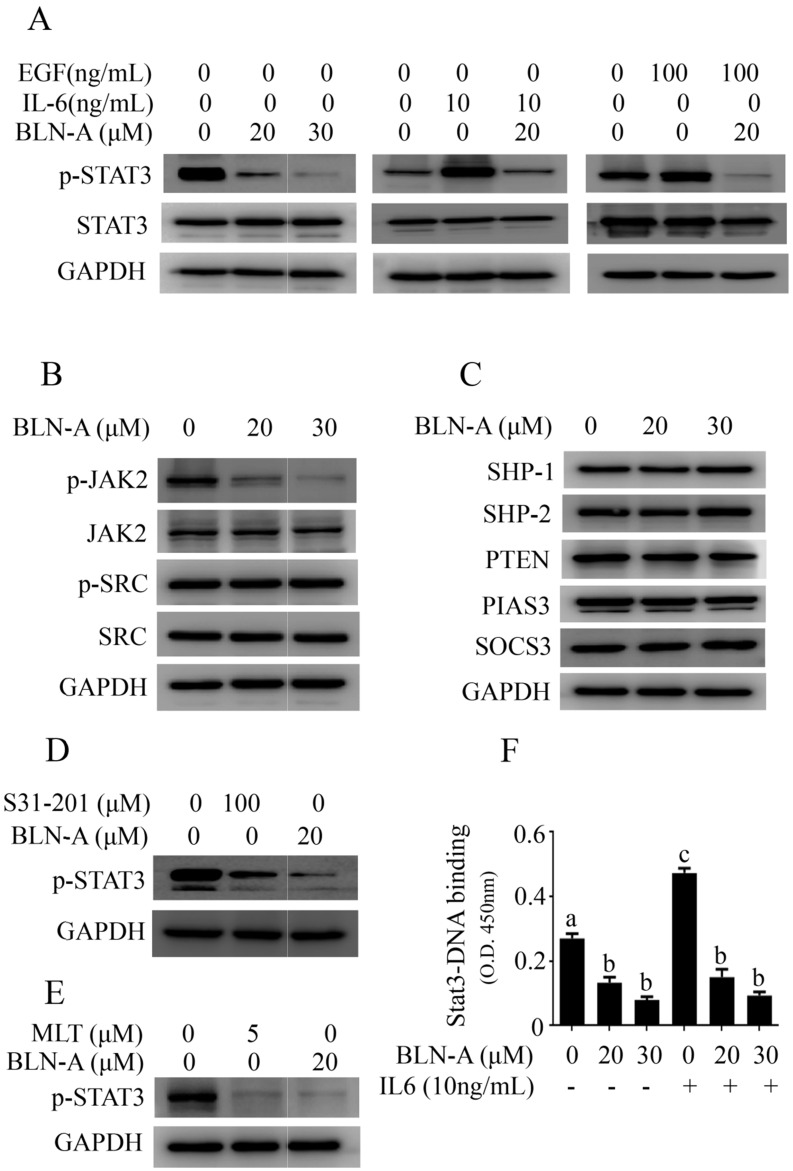
BLN-A inhibits STAT3 signaling and suppresses its DNA binding activity. **(A)** The effect of BLN-A on constitutive and inducible STAT3 activation was measured by Western blot after 4 h treatment. **(B)** Protein expression of positive regulators of STAT3 in response to BLN-A treatment for 4 h. **(C)** Immunoblotting of negative regulators of STAT3 in response to BLN-A treatment for 4 h.**(D, E)**The inhibitory effect of BLN-A on STAT3 activation was compared with commercially available STAT3 inhibitor (S31-201) and JAK inhibitor (Momelotinib) by Western blot. The quantification of protein band by image J software has been shown in supplementary Figure S2** (F)** STAT3 DNA-binding activity was measured in nuclear extracts using commercially available kit. Columns sharing the same superscript letters are not significantly (p<0.05) different from each other.

STAT3 is consistently activated in a wide range of human cancers and plays a key role in tumorigenesis, cell growth and drug resistance 7, 9, 10. Thus, we examined the effect of BLN-A on STAT3 activation. As shown in Figure 4A, BLN-A remarkably suppressed the constitutive activation of STAT3 at tyr705 dose-dependently. To discern the suppressive effects of BLN-A on inducible STAT3 activation, we used IL-6 and EGF, which are most powerful STAT3 inducers. As evident from Figure 4A**,** IL-6 (10ng/mL) and EGF (100ng/mL) treatments for 30 min powerfully increased STAT3 phosphorylation, however; this inducible STAT3 activation was effectively reversed when cells are exposed to BLN-A for 4 h prior to IL-6 and EGF treatment.

### Effect of BLN-A on STAT3 regulators

STAT3 is positively regulated by different tyrosine kinases including JAKs and Src family kinases. To get better insight into the mechanism implicated in BLN-A induced suppression of STAT3 signaling, we determined the effects of BLN-A on phosphorylation of JAK2 and Src. Our results showed that BLN-A treatment suppressed phosphorylation of JAK2 while the level of JAK2 remained unchanged. However, no suppressive activity of BLN-A was noted against Src (Figure 4B).

On the other hand, STAT3 is negatively regulated by three groups of signaling molecules: the protein tyrosine phosphatases (PTP), the protein inhibitors of activated STAT3 (PIAS) and the suppressors of cytokine signaling (SOCS) 20. To decipher the role of negative regulators of STAT3 in BLN-A induced inhibition of STAT3 signaling, we measured the expression of PIAS3, SOCS3 and various PTPs such as PTEN, SHP-1 and SHP-2. The data indicated that BLN-A failed to modulate the expression of PTPs, PIAS3 and SOCS3 (Figure 4C). The set of data indicate that BLN-A might suppress STAT3 signaling without affecting the negative regulators of STAT3. However, BLN-A has been shown to exhibit suppressive effect on JAK2 activation which could inhibit STAT3 activation.

In order to assess the potential of BLN-A as a potent STAT3 inhibitor, we compared the anti-STAT3 activity of BLN-A with S31-201 (commercially available STAT3 inhibitor) and JAK inhibitor (MLT) using Western blot. The data demonstrated that BLN-A at a concentration of 20 µM exhibited greater suppressive effect on STAT3 activation compared to five time higher concentration of S31-201 (Figure 4D). Moreover, the inhibitory effect of BLN-A at a concentration of 20 µM was comparable to that of 5 µM MLT (Figure 4E). After phosphorylation and dimerization, STAT3 acts as a transcriptional factor and regulates transcription of various target genes. To validate the suppressive effect of BLN-A on STAT3 phosphorylation, we measured STAT3 DNA binding activity. As expected, BLN-A attenuated STAT3 DNA binding activity as shown in Figure 4F.

### BLN-A directly binds with STAT3

**Figure 5:**
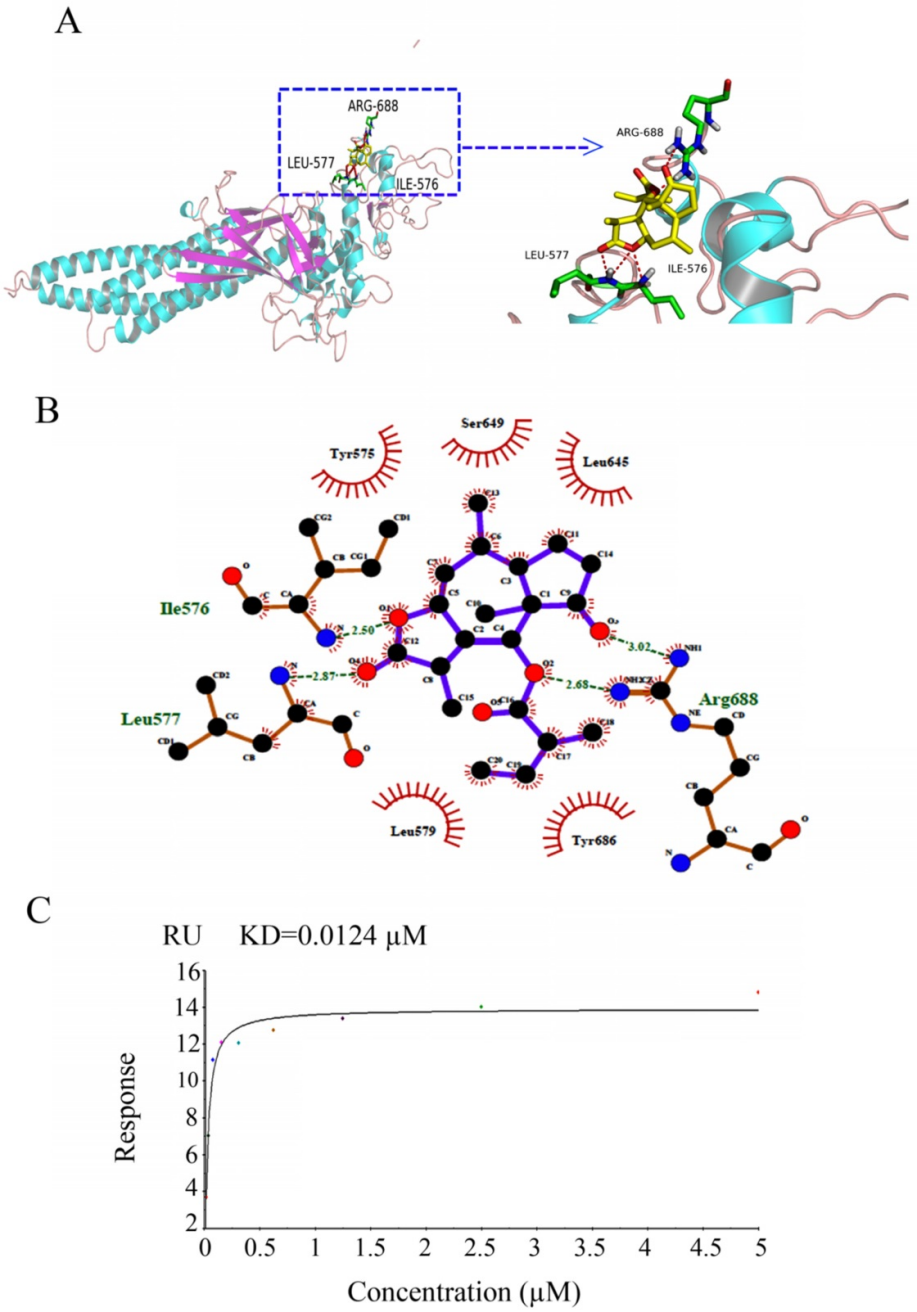
Determination of binding affinity of BLN-A with STAT3. **(A)** Hydrogen bonding between BLN-A and STAT3 was assessed by in silico docking simulation using PyMOL **(B)** Two dimensional representations of binding interactions between ligand (BLN-A) and STAT3 protein, generated by LIGPLOT. Green dotted lines indicate hydrogen bonding between BLN-A and STAT3 residues. **(C)** The binding affinity of BLN-A with STAT3 was evaluated by SPR analysis.

The binding interactions of BLN-A with STAT3 were predicted by molecular docking and verified by SPR analysis. Our docking simulation data obtained by PyMOL revealed that ARG688, LEU577 and ILE576 residues make hydrogen bonds with BLN-A (Figure 5A). Similar results were obtained from two dimensional representations of these interactions plotted by LIGPLOT. The data obtained from LIGPLOT indicate that in addition to stable hydrogen bonding between BLN-A and amino groups of ILE576, LEU577 and ARG688, hydrophobic bonding interactions of amino groups of tyr686, Ser649, Leu645, Leu579 and Tyr575 residues were observed at the periphery of BLN-A (Figure 5B). The binding interactions of BLN-A with STAT3 were further verified using SPR analysis. Figure 5C represents the RU values estimating the binding of BLN-A to immobilized STAT3 protein. We observed a dose-dependent binding between BLN-A and STAT3 with equilibrium dissociation constant (KD) about 0.0124 µM. Collective data obtained from Western blot, computational docking and SPR analysis demonstrate that BLN-A could inhibit STAT3 activation by directly binding to STAT3.

### STAT3 knockdown halts growth and augments BLN-A-induced cell death

**Figure 6:**
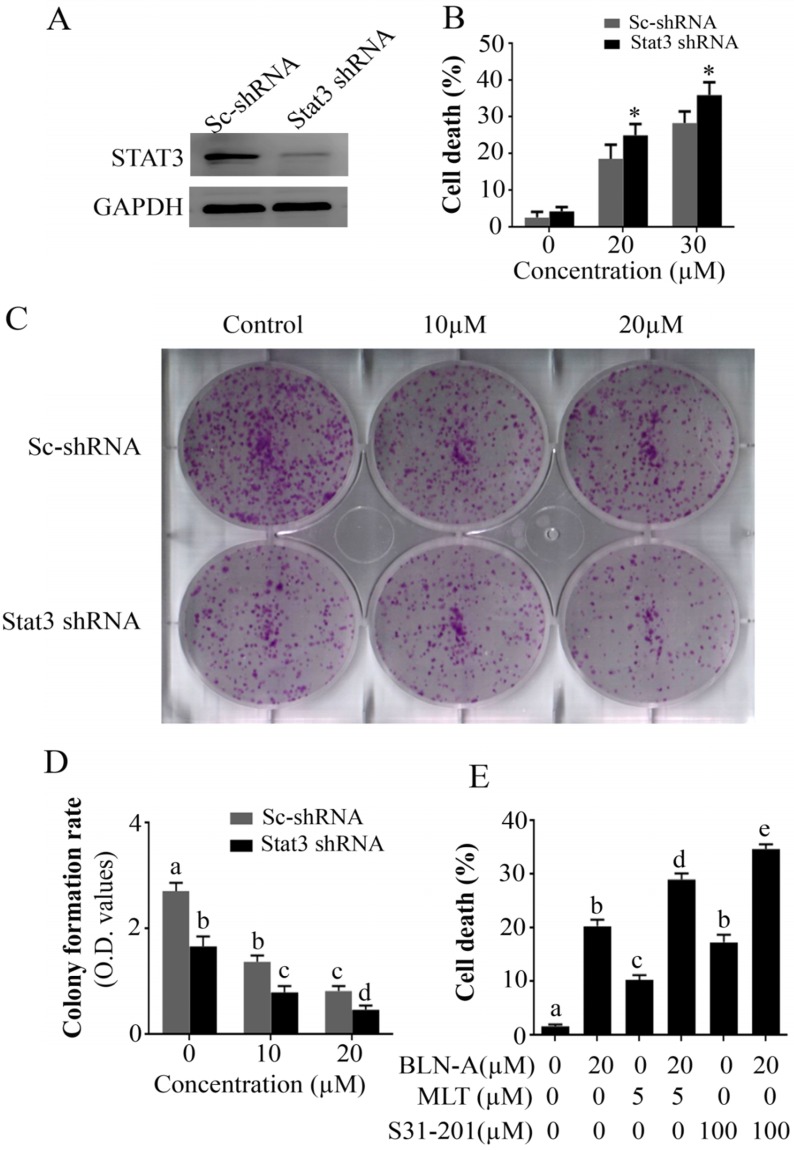
Knocking down of STAT3 halted growth and sensitizes cells to chemotherapy. **(A)** Expression of STAT3 in scrambled treated and STAT3 shRNA transfected cells was measured by immunoblotting. **(B)** TBE assay was employed to evaluate the effect of STAT3 knockdown on chemosensitization. (**C, D**) Effect of STAT3 knockdown on colony formation in BLN-A treated and un-treated cells. (E) Effect of combine treatments of BLN-A with S31-201 and Momelotinib on cell death.

To describe the functional role of STAT3 in A549 cell death and proliferation, we used STAT3 knockdown A549 cell line and compared the effect of BLN-A treatment in parental A549 cells and STAT3 knockdown A549 cells using TBE assay and colony forming assay. The expression of STAT3 in A549 and STAT3 knockdown A549 cell lines has been shown in Figure 6A. We found that STAT3 knockdown remarkably improved the anticancer efficacy of BLN-A (Figure 6B-D). To further validate the role of STAT3 in A549 cell death, we determined the efficacy of S31-201 and MLT as a single agent and/or in combination with BLN-A by TBE assay. Our results showed that S31-201, MLT and BLN-A significantly increased cell death in A549 cells as a single agent while additive effects were noted when S31-201 and MLT were combined with BLN-A (Figure 6E). Collective data from aforementioned assays demonstrate clearly that STAT3 is implicated in cell proliferation and survival.

## Discussion

In this study, we demonstrated that BLN-A inhibits STAT3 activation and induces apoptosis in A549 NSCLC. BLN-A has induced apoptosis by interfering with redox balance and STAT3 signaling. The mechanism underlying the BLN-A-induced apoptosis has been associated with suppression of STAT3 activation, ROS generation, GSH depletion, mitochondrial dysfunction, and cleavage of caspase-3 and PARP. Moreover, we have explored the in-depth mechanism by which BLN-A suppresses STAT3 activation. Induction of mitochondrial apoptosis by BLN-A has previously been reported by You et al. in colon adenocarcinoma cells via inhibition of PI3K/AKT/mTOR signaling pathway 16. Likewise, we have previously reported that BLN-A induces apoptosis in U87 glioblastoma cells via induction of oxidative stress and activation of intrinsic apoptotic pathway 14. However, in both these studies, the functional role of ROS in apoptosis induction remained unknown. In the present study, we have defined the role of BLN-A induced ROS in suppression of growth and induction of apoptosis in lung adenocarcinoma cells. Scavenging ROS by NAC protected the cells from cytotoxic effects of BLN-A by reversing the effect of BLN-A on various cellular targets linked to growth arrest and apoptosis. The notion stemming from these results demonstrates that BLN-A triggers apoptosis primarily by inducing ROS generation and depleting GSH in lung cancer cells.

It is well established now that induction of ROS generation is the primary event in anticancer activity of sesquiterpene lactones which is mediated by conjugation of alkylating centers with cysteine-containing biomolecules 21-23. These alkylating centers readily react with reduced GSH and thereby increase ROS generation by disrupting redox homeostasis of the cell which ultimately leads to mitochondrial damage and initiation of intrinsic apoptosis 14. In line with other sesquiterpene lactones 24, BLN-A induced mitochondrial apoptosis via ROS generation as evident from MMP dissipation, decreased Bcl-2/bax ratio and cleavage of caspase-3 and PARP, which are considered the classical markers of apoptosis 25.

Secondly, we probed the mechanism by which BLN-A suppresses STAT3 activation. STAT3 activation is controlled through multiple mechanisms including cytokine receptors, RTKs, n-RTKs, PTPs, SOCS and PIAS 26. IL-6 and EGF are the most common ligands which upon binding with corresponding receptor tyrosine kinase, induce activation of n-RTKs such as JAK2 and Src. Tyrosine kinase activity of JAK2 and Src leads to activation of STAT3 at tyr705 27. Once activated, STAT3 is translocated from cytosol into the nucleus where it induces transcription of various downstream target genes important for tumor induction and progression 28-31. Here in this study, we found that BLN-A inhibited constitutive as well as IL-6- and EGF-induced STAT3 activation at tyr705. Consistent with suppression of STAT3 phosphorylation at tyr705, BLN-A decreased STAT3 DNA-binding activity. Chen et al. have previously reported that BLN-A inhibits STAT3 activation via blocking JAK-2 activation 17. Consistent with the previous report, we found similar inhibitory effect of BLN-A on JAK-2 activation, however; no effect on Src activation was noted. To decipher the exact mechanism of inhibition of STAT3 activation, we further extended our study to investigate the effect of BLN-A on negative regulators of STAT3 signaling.

STAT3 activation is negatively regulated by three families of signaling molecules; PTPs, SOCS and PIAS 32. PTPs inhibit STAT3 activation by directly dephosphorylating RTKs, JAKs and STAT3 28. Therefore, we determined the protein expression of various PTPs involved in negative regulation of STAT3. The expression of PTEN, SHP-1, and SHP-2 remained unchanged in BLN-A treated cells indicating that BLN-A suppresses STAT3 activation without affecting PTPs. The findings are further supported by previous reports showing inhibition of STAT3 activation by alantolactone, a sesquiterpene lactone without the involvement of PTPs in breast and lung cancer cells 2, 28. SOCS proteins negatively regulate STAT3 activation by inhibiting JAK kinase or by shielding the STAT3 binding site on the cytokine receptors. Among SOCS, SOCS3 is the best characterized in regulation of JAK-STAT3 signaling so far 26. Like PTPs, the expression of SOCS3 remained unaffected in BLN-A treated cells. PIAS have been reported to suppress STAT3 signaling by inhibiting STAT3 DNA-binding activity 9. We also measured the expression of PIAS3 which is considered a specific repressor of STAT3 transcriptional activity. However, the expression of PIAS3 was not affected by BLN-A. The collective data demonstrate that BLN-A induces inhibition of STAT3 activation by a mechanism that does not involve modulation of PTPs, SOC3 and PIAS3. Finally we hypothesized that BLN-A could directly bind with STAT3 and thereby inhibits its activation. To support our hypothesis, we performed computational docking. Our docking study disclosed that BLN-A is predicted to bind with STAT3. To further confirm the binding of BLN-A to STAT3, we performed SPR analysis. Our SPR analysis confirmed a dose-dependent binding affinity of BLN-A with STAT3. Collective data obtained from Western blot, computational docking and SPR analysis indicate that BLN-A could inhibit STAT3 activation via two main mechanisms including direct binding and inhibition of JAK-2 activation, at least to the extent of this study.

Compelling evidence from a plethora of published research reports suggest that STAT3 is implicated in cancer cell survival and suppression of STAT3 activation could effectively inhibit cell proliferation and promote apoptosis in multiple human cancers 1,2,28,33. Our data generated by knockdown of STAT3 by shRNA confirmed the pro-survival effect of STAT3. STAT3 knockdown by shRNA suppressed growth and potentiated BLN-A mediated cell death as evident from the data obtained from colony formation and TBE assays. Moreover, combine treatment of BLN-A with S31-201 (STAT3 inhibitor) and MLT (JAK inhibitor) significantly enhanced cell death compared to either treatment alone, further supporting the functional role of STAT3 in BLN-A mediated apoptosis.

**Figure 7:**
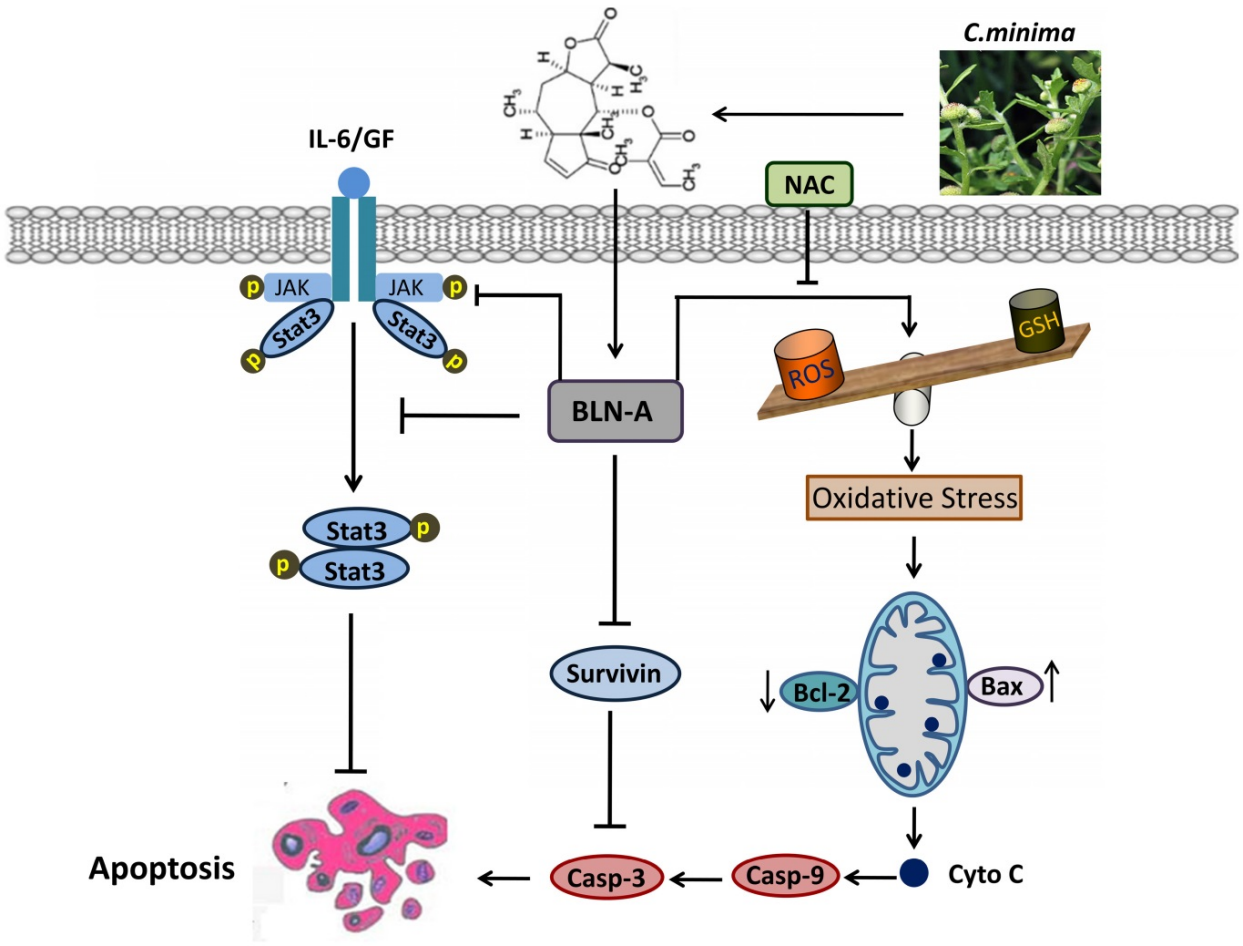
A schematic model representing the cellular targets and anticancer mechanism of BLN-A in A549 lung adenocarcinoma cells.

In conclusion, we have demonstrated that BLN-A induces apoptosis by promoting ROS generation and inhibiting STAT3 activation. Moreover, we have provided in-depth mechanism of BLN-A mediated suppression of STAT3 activation and functional role of STAT3 inhibition in BLN-A mediated apoptosis. Our findings suggest that BLN-A may be developed into a novel STAT3 inhibitor and as a potential therapeutic agent against lung adenocarcinoma. However, further *in vivo* study is needed to evaluate the anticancer activity of BLN-A to validate it as an attractive antitumor agent for clinical trials. A schematic model for the anticancer mechanism of BLN-A in A549 lung cancer cells has been shown in Figure 7.

## Supplementary Material

Supplementary figures.
