# Supplementary material for: Brevilin A induces ROS-dependent apoptosis and suppresses STAT3 activation by direct binding in human lung cancer cells
Source: J Cancer. 2020 Apr 6;11(13):3725–35. doi: 10.7150/jca.40983 (PMC7171504; doi:10.7150/jca.40983)
Supplement: Supplementary file 1 — Supplementary figures. [file jcav11p3725s1.pdf]

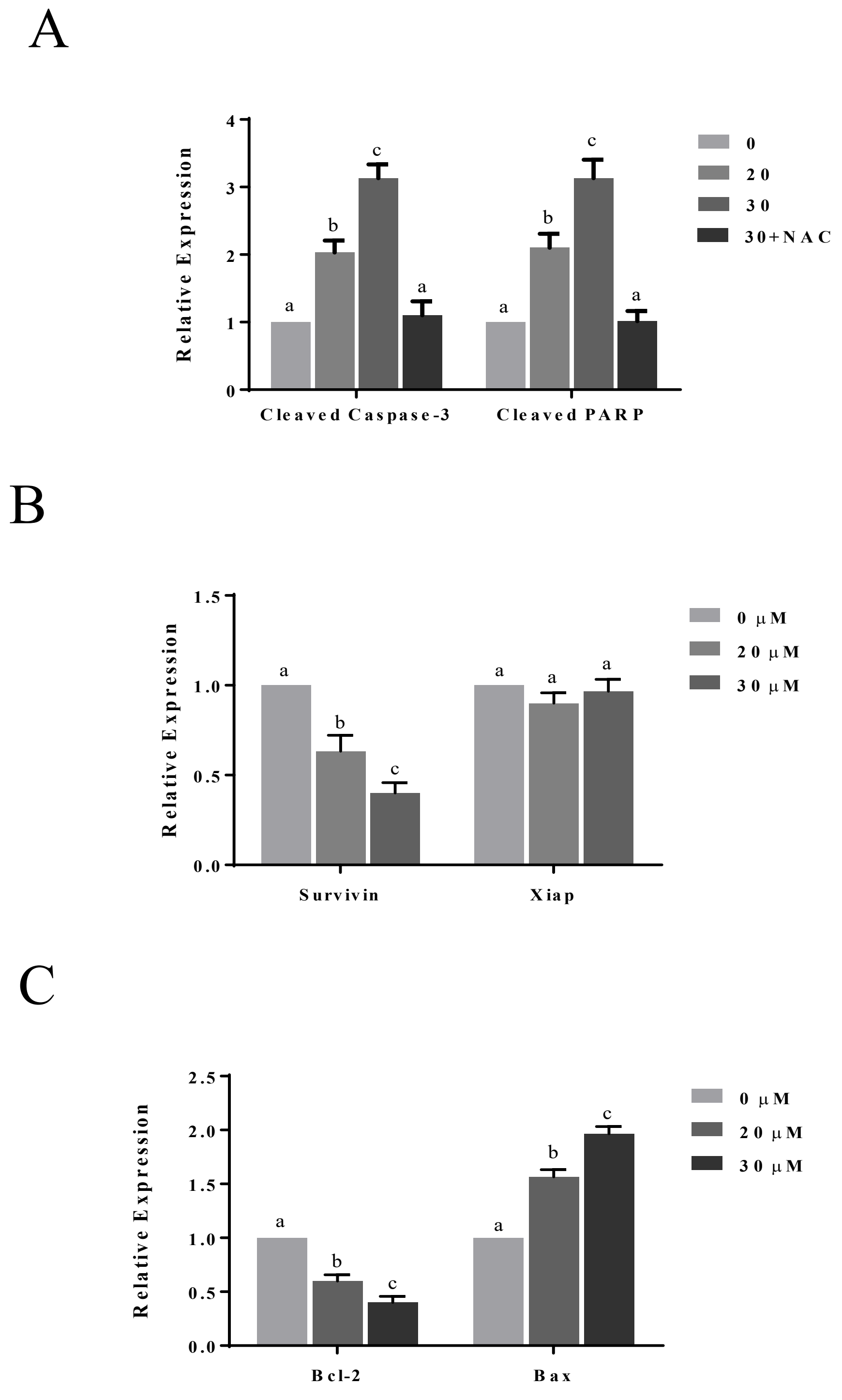

Figure S1: Quantification of Western blot images presented in Figure 2 and 3. **(A)** Quantification of data presented in figure 2C. **(B)** Quantification of data presented in figure 2D. (C) Quantification of immunoblots presented in figure 3D. Image J software was used to quantify protein bands from 3 repeated experiments. The bars with different superscript letters differ significantly ($p<0.05$).

A

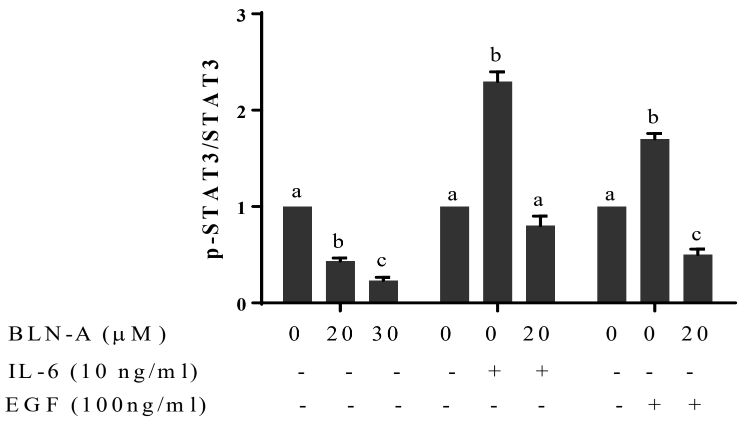
p-STAT3/STAT3
3
2
1
0
a
b
c
a
b
a
a
b
c
BLN-A (μM)
0 20 30 0 0 20 0 0 20
IL-6 (10 ng/ml)
- - - - + + - - -
EGF (100ng/ml)
- - - - - - - + +

B

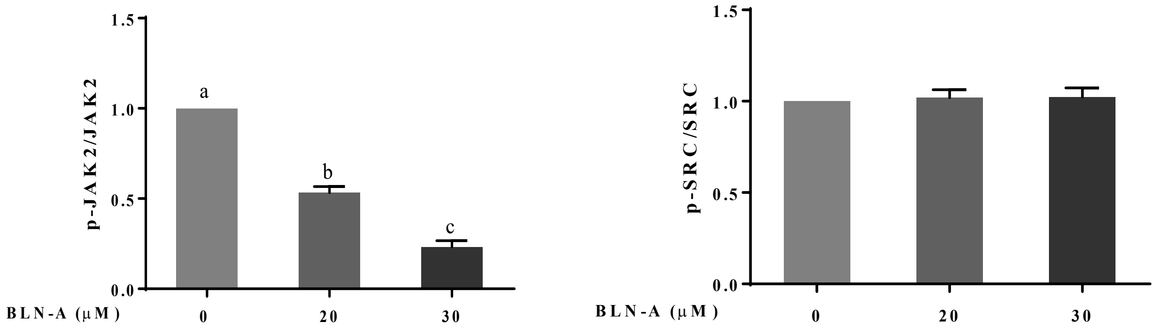
p-JAK2/JAK2
1.5
1.0
0.5
0.0
a
b
c
BLN-A (μM)
0
20
30
p-SRC/SRC
1.5
1.0
0.5
0.0
BLN-A (μM)
0
20
30

C

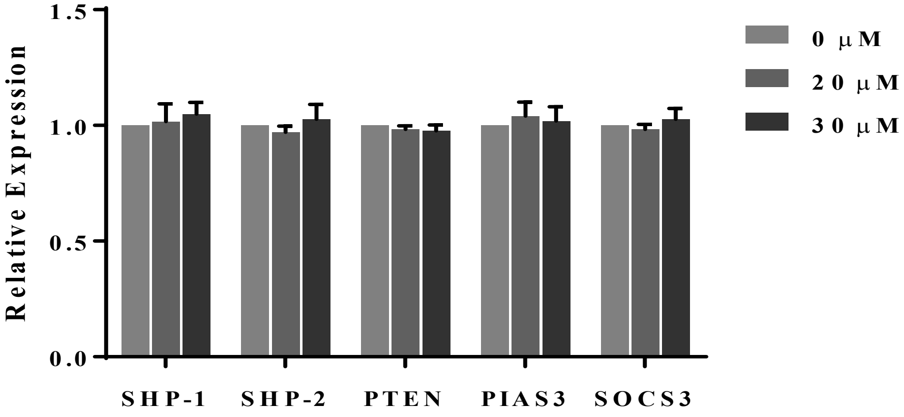
Relative Expression
1.5
1.0
0.5
0.0
0 μM
20 μM
30 μM
SHP-1
SHP-2
PTEN
PIAS3
SOCS3

D

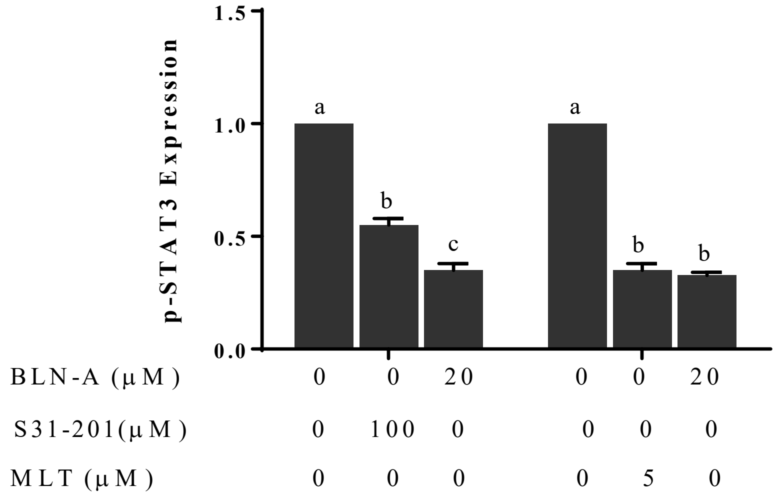

Figure S2: Quantification of Western blot images presented in figure 4. (A) Quantification of data presented in figure 4A of the manuscript. The graph bars which share different superscript letter are significantly different at $p<0.05$. **(B)** Quantification of p-JAK2 and p-SRC presented in figure in figure 4B of manuscript. P-STAT3, p-JAK2 and p-SRC were normalized to total STAT3, JAK2 and SRC, respectively. **(C)** Quantification of expression of various proteins presented in figure 4C. **(D)** Quantification of protein band presented in figure 4D and E. The bars which do not share same superscript letter differ significantly at $p<0.05$.
